# The Use of a Two-Tiered Testing Strategy for the Simultaneous Detection of Small *EGFR* Mutations and *EGFR* Amplification in Lung Cancer

**DOI:** 10.1371/journal.pone.0117983

**Published:** 2015-02-26

**Authors:** Marzena Anna Lewandowska, Karol Czubak, Katarzyna Klonowska, Wojciech Jozwicki, Janusz Kowalewski, Piotr Kozlowski

**Affiliations:** 1 Molecular Oncology and Genetics Unit, Department of Tumour Pathology and Pathomorphology, Franciszek Lukaszczyk Oncology Centre, Bydgoszcz, Poland; 2 Department of Thoracic Surgery and Tumours, Ludwik Rydygier Collegium Medicum, Nicolaus Copernicus University, Bydgoszcz, Poland; 3 European Centre of Bioinformatics and Genomics, Institute of Bioorganic Chemistry, Polish Academy of Sciences, Poznan, Poland; 4 Poznan University of Technology, Pl. Marii Sklodowskiej-Curie 5, 60-965, Poznan, Poland; 5 Department of Tumour Pathology and Pathomorphology, Franciszek Łukaszczyk Oncology Centre, Ludwik Rydygier Collegium Medicum, Nicolaus Copernicus University, Bydgoszcz, Poland; 6 Department of Thoracic Surgery and Tumours, Franciszek Łukaszczyk Oncology Centre, Ludwik Rydygier Collegium Medicum, Nicolaus Copernicus University, Bydgoszcz, Poland; Catalan Institute of Oncology, SPAIN

## Abstract

Lung cancer is the leading cause of cancer-related death worldwide. Recent progress in lung cancer diagnosis and treatment has been achieved due to a better understanding the molecular mechanisms of the disease and the identification of biomarkers that allow more specific cancer treatments. One of the best known examples of personalized therapy is the use of tyrosine kinase inhibitors, such as gefitinib and erlotinib, for the successful treatment of non-small-cell lung cancer patients selected based on the specific *EGFR* mutations. Therefore, the reliable detection of mutations is critical for the application of appropriate therapy. In this study, we tested a two-tiered mutation detection strategy using real-time PCR assays as a well-validated high-sensitivity method and multiplex ligation-dependent probe amplification (MLPA)-based EGFRmut+ assay as a second-tier standard-sensitivity method. One additional advantage of the applied MLPA method is that it allows the simultaneous detection of EGFR mutations and copy-number alterations (i.e., amplifications) in *EGFR, MET* and *ERBB2*. Our analysis showed high concordance between these two methods. With the use of this two-tier strategy, we reliably determined the frequency of *EGFR* mutations and *EGFR, MET* and *ERBB2* amplifications in over 200 lung cancer samples. Additionally, taking advantage of simultaneous copy number and small mutation analyses, we showed a very strong correlation between EGFR mutations and EGFR amplifications and a mutual exclusiveness of EGFR mutations/amplifications with MET and ERBB2 amplifications. Our results proved the reliability and usefulness of the two-tiered EGFR testing strategy.

## Introduction

Lung cancer is the leading cause of cancer-related death worldwide. The treatment of lung cancer is traditionally based on a histopathological evaluation that distinguishes two major types of lung cancer: small-cell lung cancer (SCLC) and non-small-cell lung cancer (NSCLC), the latter of which can be subdivided into squamous cell carcinoma, large cell carcinoma, adenocarcinoma and cancers with mixed histology. Substantial recent progress in the treatment of lung cancer (especially adenocarcinomas) has been achieved by advances in our understanding of its pathology; the current treatment options include specialized agents based on the presence or absence of specific genetic biomarkers (“personalized therapy”), such as mutations in the epidermal growth factor receptor (*EGFR*) [1] or gain-of-function translocations or inversions involving the anaplastic lymphoma receptor tyrosine kinase (*ALK*) [2].

It was shown that certain somatic mutations within the kinase domain of *EGFR* sensitize cancers to treatment with *EGFR*-specific tyrosine kinase inhibitors (TKIs), such as erlotinib or gefitinib (reviewed in [3]). Among the most common sensitizing *EGFR* mutations are L858R in exon 21 and in-frame deletions in exon 19, which together account for over 80–85% of all *EGFR* mutations. However, the occurrence of the secondary T790M mutation in exon 20 causes acquired resistance to TKIs and causes the progression of cancers treated with TKIs [4,5]. Therefore, the reliable detection of *EGFR* mutations is an important factor that allows the personalized treatment of lung cancer patients.

In the last 10 years, numerous methods with different sensitivities and specificities have been used to detect *EGFR* mutations in cancer samples. These methods include Sanger sequencing, single strand conformation polymorphism (SSCP) [6], co-amplification at lower denaturation temperature-PCR (COLD PCR) [7], immunohistochemistry with *EGFR*-mutation specific antibodies [8], peptide nucleic acid-locked nucleic acid (PNA-LNA) PCR clamp assays, real-time PCR (RT-PCR) methods [9] and next generation sequencing [10]. However, none of the methods that have been used so far are ideal, and each of these methods has limitations that mostly relate to the following characteristics of cancer samples: (i) diversified types of tumor samples available for analysis (surgical, biopsied), (ii) contamination with normal cells, (iii) the genetic heterogeneity of tumors, (v) the often low frequency of the analyzed mutations, (iv) degradation of DNA, and (v) damage to or modification of DNA [the two latter factors also apply to formalin-fixed, paraffin-embedded (FFPE) samples, which are the most frequently available samples]. The most serious problems resulting from the limitations of tumor sample analysis are false negative and false positive errors that may lead to the misclassification and inadequate treatment of cancer. Therefore, to reduce the fraction of misclassified samples, it was recently proposed in the evidence-based guideline of three professional societies (College of American Pathologists, International Associations for the Study of Lung Cancer, and Association for Molecular Pathology) that, if possible, *EGFR* mutation testing should be carried out with two methods (a two-tiered testing strategy). This guideline represents state-of-the-art molecular lung cancer testing and was jointly published in three journals [11–13]. For simplicity we will subsequently refer only to [11]. This two-tier method should be based on different mutation detection principles and should cover different ranges of sensitivity, consisting of standard-sensitivity and high-sensitivity methods.

In this study, we tested over 200 NSCLC samples with the use of two complementary methods, a routinely used commercial RT-PCR assay (a high-sensitivity method) [9] and a new multiplex ligation-dependent probe amplification (MLPA)-based EGFRmut+ assay (a standard-sensitivity, second-tier method) [14]. Our analysis showed a high concordance between these two methods and thus proved the reliability and usefulness of the EGFRmut+ assay as a second-tier method for *EGFR* mutation testing. With the use of these methods, we characterized and estimated the frequency of somatic *EGFR* mutations in a set of lung cancer samples from central Poland. One additional advantage of the EGFRmut+ assay is that it allows a mutation analysis and relative copy number determination (i.e., amplification detection) in parallel. We used this approach to find a very strong correlation between *EGFR* amplification and the occurrence of *EGFR* mutations and to determine the rough frequency of mutant alleles in our analyzed samples.

## Materials and Methods

### Selection and processing of NSCLC samples for molecular analysis

We retrospectively reviewed a cohort of 239 patients with histopathologically confirmed NSCLC diagnosed from 2011 to 2012 at the Franciszek Lukaszczyk Oncology Center in Bydgoszcz (central Poland). The age of the patients ranged from 35 to 81. A total of 239 specimens that passed the quality control steps (microscopic analysis and tumor content qualification as well as qualitative and quantitative DNA analysis) were obtained following 143 surgeries, 91 fine-needle aspiration (FNA) procedures, and 5 endobronchial ultrasound with guided transbronchial needle aspiration (EBUS-TBNA) procedures or pleural fluid samplings. The samples were stained with hematoxylin and eosin for the qualitative and quantitative analysis of tumor cells in the analyzed material (including macrodissection in marked out samples). The qualification of biological material for molecular analysis was based on the previously described qualitative and quantitative scales for cytological [9] and histological [15] material. Informed written consent for genetic testing, approved by the F. Lukaszczyk Oncology Center in Bydgoszcz was obtained from all of the patients and the study was approved by our local ethics committee, Bioethics Committee of Ludwig Rydygier Collegium Medicum in Bydgoszcz, Nicolaus Copernicus University in Torun. The data were analyzed anonymously.

### DNA isolation

DNA isolation was performed after the macrodissection of a region indicated by the pathomorphologist. Genomic DNA was isolated from FFPE adenocarcinoma tissue or cytological smears using a QIAamp FFPE Mini Kit (QIAGEN) according to the manufacturer’s instructions with the following modifications. We resuspended the pellet in 180 μl of Tissue Lysis Buffer with 20 μl of proteinase K, and then vortexed and continuously shook the cell pellet at short intervals during an overnight incubation at 56°C. The DNA quantity and quality were evaluated by NanoDrop absorbance analysis and agarose gel electrophoresis.

### Mutation analysis by Real-Time PCR

The RT-PCR method uses mutation-specific probes to evaluate 29 point mutations, including the T790M mutation (EGFR-RT52; Entrogen, Inc., Tarzana, CA). A DNA quantity of 200–650 ng was adequate for the detection of 29 mutations in the samples of interest; the internal control VIC fluorescent probes and *EGFR* fluorescent probes were FAM dye-labeled. The amplification curves were evaluated according to the recommendations of the manufacturer.

### Mutation and amplification analysis by MLPA

MLPA analysis was performed with the use of a custom-designed EGFRmut+ assay, which has previously been described in greater detail [14]. This assay simultaneously allows the detection of oncogenic *EGFR* mutations and an analysis of the copy number (amplification detection) of *EGFR*, *MET*, and *ERBB2*. All reagents except the EGFRmut+ probe mix were purchased form MRC-Holland Amsterdam, The Netherlands (www.mlpa.com). The MLPA reactions were run according to the manufacturer’s general recommendations (MRC-Holland), as described earlier in [16,17]. Briefly, 5 μl of genomic DNA (at a concentration of approximately 20 ng/nl) was incubated at 98°C for 5 min, cooled to room temperature and mixed with 1.5 μl of EGFRmut+ probes mix and 1.5 μl of SALSA hybridization buffer. The reaction was then denatured at 95°C for 2 min and hybridized at 60°C for 16 h. The hybridized probes were ligated at 54°C for 15 min by the addition of 32 μl of ligation mixture. Following heat inactivation, the ligation reaction was cooled to room temperature, mixed with 10 μl of PCR mixture (polymerase, dNTPs, and universal primers, one of which was labeled with fluorescein) and subjected to PCR amplification for 35 cycles. The MLPA products were subsequently diluted 20x in HiDi formamide containing GS Liz600, which was used as a DNA sizing standard, and separated via capillary electrophoresis (POP7 polymer) in an ABI Prism 3130XL apparatus (Applied Biosystems). The obtained electropherograms were analyzed using GeneMarker software v1.91 (2.4.0). The signal intensities (peak heights) were retrieved and transferred to prepared Excel sheets (available upon request). For each individual sample, the signal intensity of each probe was divided by the average signal intensity of the control probes to normalize the obtained values and to equalize run-to-run variation, and the normalized value for each peak was then divided by a corresponding value in the reference samples and multiplied by 2. The final MLPA result of each sample is presented on bar-plot, in which the bars show the relative copy number value of the subsequent probes.

### EGFR copy number analysis by quantitative PCR (qPCR) and droplet digital PCR (ddPCR)

The qPCR analysis was performed with the use of MESA GREEN qPCR MasterMix Plus for SYBR Assay (Eurogentec, Seraing, Belgium), according to the manufacturer’s general recommendations. ddPCR was performed with the use of QX200 system and EvaGreen Supermix (BIO-RAD, CA, USA) according to manufacturer’s general recommendations, as described before [18,19]. The ddPCR analysis was performed in a multiplex format with the co-amplification of control-amplicon and one of the test-amplicons in one reaction. To achieve proper separation of droplet types, the intensity of test and control signal was differentiated by amplicons’ length and primers’ concentrations. For both methods the same set of PCR primers was used: (i) test-amplicon for *EGFR* exon 2: forward primer GCAGTTGGGCACTTTTGAAG, reverse primer TTCCAAATTCCCAAGGACCA (concentration in qPCR—300 nM, concentration in ddPCR—100 nM, amplicon length 83 bp); (ii) test-amplicon for EGFR exon 18: forward primer TTGTGGAGCCTCTTACACCC, reverse primer CCTTCAAGATCCTCAAGAGAGC (concentration in qPCR—300 nM, concentration in ddPCR—100 nM, amplicon length 64 bp); (iii) control-amplicon: forward primer GCTGACCTGTTGGCTGAAAA, reverse primer GAATCGCTGTGGCCTTGATG (concentration in qPCR—300 nM, concentration in ddPCR—200 nM; amplicon length 113 bp). The amplicons either overlapped, or were closely located to the following MLPA probes: EGFR_e2, EGFR_e18, and ctrl_1, respectively. The optimized annealing temperature was set at 59°C and 58°C, in qPCR and ddPCR, respectively.

## Results

**Fig 1:**
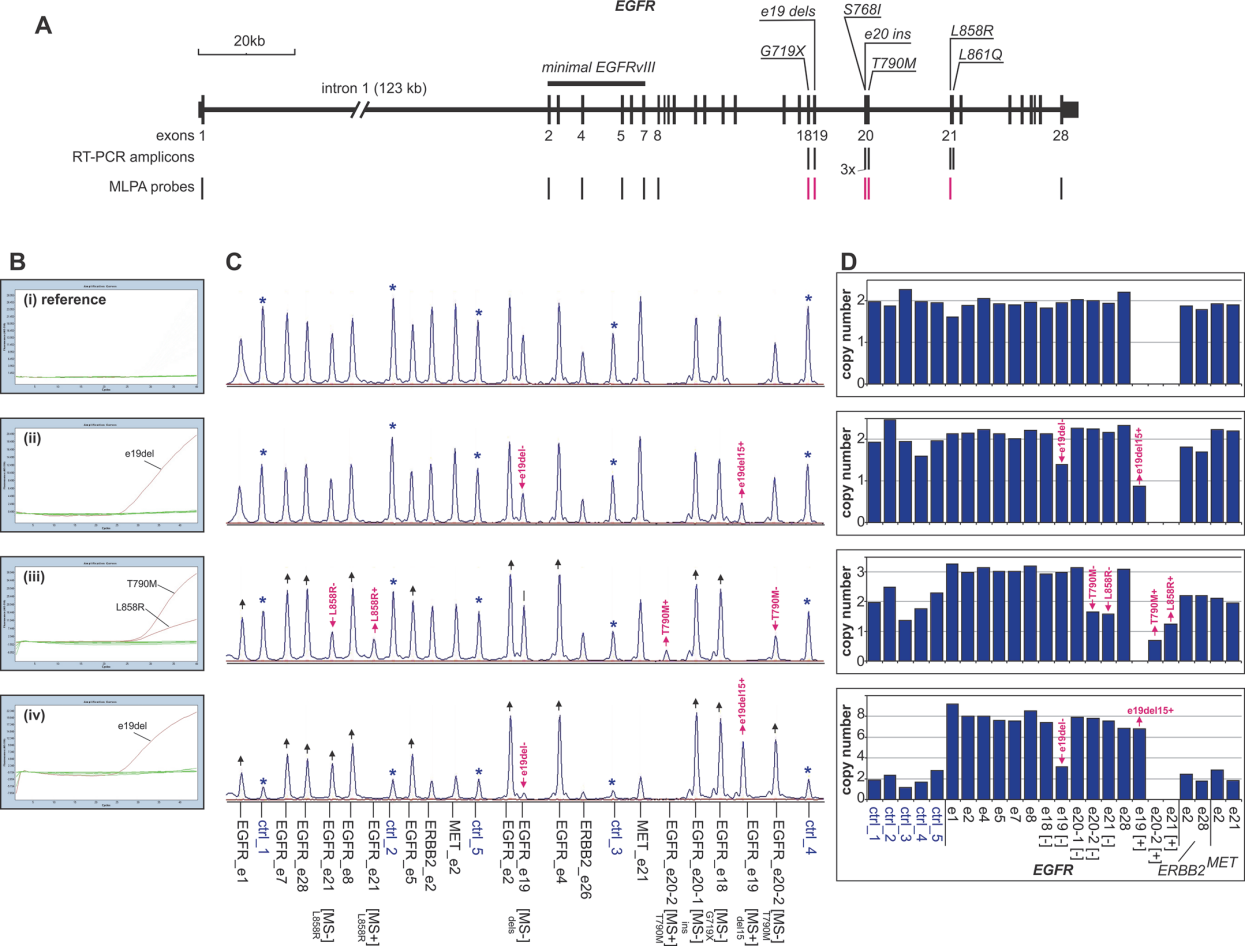
The strategy of *EGFR* mutation detection by combined RT-PCR and MLPA-based analyses. A) Map of the *EGFR* gene with the positions of the RT-PCR amplicons (EGFR-RT52) and MLPA probes (EGFRmut+ assay) indicated (vertical lines under the map). The mutation-sensitive EGFRmut+ probes are indicated in red. The positions of oncogenic *EGFR* mutations are indicated over the map. B) The RT-PCR results representing (from the top) (i) the reference sample (a sample with no mutations or amplification), (ii) a sample with the most common in-frame deletion in exon 19 (c.2235_2249del15), (iii) a sample with both L858R in exon 21 and T790M in exon 20, and (iv) a sample with an in-frame deletion in exon 19 (c.2235_2249del15) and *EGFR* amplification. In each graph, the overlapping results of the 8 RT-PCR reactions covering 29 *EGFR* mutations are shown. The red lines indicate the positive amplification-curves of specific *EGFR* mutations (pointed on graph), and the green base line represents the non-amplified signal due to the lack of the evaluated mutation in the analyzed sample. C) MLPA electropherograms of samples analyzed by RT-PCR (panel B). The probe IDs are indicated under the electropherograms. The asterisks indicate the control probes; the pink arrowheads indicate reduced signal of MS- probes and increased signal of MS+ probes, respectively; and the black arrowheads indicate amplified signals of *EGFR*-specific probes. D) Bar plots corresponding to the electropherograms shown in panel C and representing the normalized copy number value (y-axis) of each probe (x-axis). The pink arrowheads indicate a reduced copy number value of the MS- probes and an increased copy number value of the MS+ probes, respectively.

All 239 samples were analyzed as blind samples by two methods: (i) a commercial RT-PCR assay (EGFR-RT52; Entrogen Inc.) that covers 29 of the most common oncogenic mutations in exons 18, 19, 20 and 21 of *EGFR* (for details, see the manufacturers webpage; http://www.entrogen.com/web2/egfr-mutation-analysis-kit) and (ii) a custom-designed MLPA assay (EGFRmut+) that simultaneously allows the detection of amplifications and small mutations in *EGFR* (for details see [14]) (Fig. 1). Briefly, in addition to the standard dosage-sensitive (DS) probes, the EGFRmut+ assay is also composed of two types of mutation-sensitive probes. Mutation-sensitive (MS-) probes are types of DS probes that are specific to the wild-type sequence but overlap with the sites of the following mutations: G719A/S/C (G719X) in exon 18 (probe e18), in-frame deletions in exon 19 (probe e19), S768I and in-frame insertions in exon 20 (probe e20-1), T790M in exon 20 (probe e20-2) and L858R in exon 21 (probe e21). The occurrence of one of these mutations in an analyzed sample causes a decrease in the signal of the corresponding MS- probe. The EGFRmut+ assay also contains three mutation-sensitive (MS+) probes that are specific for mutant sequences. The signals from these probes occur only if the corresponding mutation is present. Two of these probes recognize the sequences of the most common *EGFR* mutations (the most common in-frame deletion in exon 19, c.2235_2249del15 (probe e19+) and L858R (probe e21+)), and the third recognizes the T790M mutation, which is associated with TKI resistance (probe e20-2+). Additionally, EGFRmut+ contains a few DS probes that are specific either for *MET* or *ERBB2* that allow amplifications of these genes to be detected.

### Characteristics of the detected mutations

In total, we detected 30 mutations in 29 out of 239 samples (12.1%). Both L858R and T790M were found in one sample. Among the identified mutations were 16 in-frame deletions in exon 19 (53%), 9 substitutions L858R (30%), 2 substitutions L861Q (7%), one substitution G719X, one in-frame insertion in exon 20 and one substitution T790M (S1 Table; summarized in Table 1). The mutations were much more frequent in women 22/68 than in men 7/142 (24.4% versus 4.7%, respectively; p<0.0001). A stratification of the mutation frequency by age [<55 (13%), >55 (11.9%)] and sample type [FFPE (11.9%), cytological samples (12.5%)] did not show significant influences of these factors on mutation frequency (Table 1). We also did not observe a decrease in the mutation frequency in samples with a lower percentage of tumor cells (PTC). Note however, that only a small fraction (roughly 5%) of the analyzed samples had PTC≤30%.

**Table 1:** Sample characteristics and mutations detected by combined RT-PCR and MLPA-based analyses.

general statistics				types of *EGFR* mutations						gains + amplifications (%)		
		number of samples	samples with *EGFR* mut. (%)	G719X	in-frame dels in ex19 (*)	in-frame ins in ex20	L858R	L858R+ T790M	L861Q	EGFR	ERBB2	MET
	all patients	239	29 (12.1)	1†	16 (10)	1	8	1	2‡	19 (7.9)	5 (2.1)	28 (11.7)
sex	female	90	22 (24.4)	1	11 (7)	0	7	1	2	16 (17.8)	1 (1.1)	12 (13.3)
	male	149	7 (4.7)		5 (3)	1	1			3 (2.0)	4 (2.7)	16 (10.7)
age	35–55	54	7 (13.0)		4 (2)	1	1	1	2	6 (11.1)		6 (11.1)
	56–81	185	22 (11.9)	1	12 (8)		7			13 (7.0)	5 (2.7)	22 (11.9)
type of material	FFPE samples	143	17 (11.9)	1	9 (4)		5		2	8 (5.6)	2 (1.4)	20 (14)
	cytological samples §	96	12 (12.5)		7 (6)	1	3	1		11 (11.5)	3 (3.1)	8 (8.3)

* the most frequent in-frame deletion in exon 19 (c.2235_2249del15)

†, ‡ mutations that were not detected by MLPA-based assay due to low PTC and the lack of mutation-specific probe, respectively

§ includes FNA, EBUS-TBNA and pleural fluid sampling.

### Comparison of RT-PCR and MLPA mutation detection methods

There is no gold standard method for mutation detection in cancer samples, where a particular mutation may account for a very low fraction of the analyzed DNA. Therefore, to evaluate the quality of the MLPA-based EGFRmut+ assay, we compared it to the routinely used and well validated RT-PCR method. A direct comparison of the results of RT-PCR and MLPA showed a very high concordance between these two methods (98.7% concordant results) as well as 100% specificity (no false positives) and 90% sensitivity (27 out of 30 mutations detected) of the EGFRmut+ test. Among the 3 mutations that were not detected by the EGFRmut+ assay were two cases with the L861Q substitution, which is not covered by EGFRmut+ probes (Fig. 1A), and one G719X mutation in a sample with a low PTC (15%). Additionally, among the samples analyzed by MLPA were 4 blind duplicates with 3 different mutations. In all cases, the results of the duplicate samples were concordant. Moreover, it must be noted that MS+ probes (signal occurrence) detect mutations more easily and with a higher confidence than MS- probes (signal decrease). The MS+ probes allowed the confident detection of mutations, even when those mutations account for a very small fraction of the analyzed DNA (∼10%) in samples with PTC≤30%. For example, the L858R mutation, which is covered by both MS+ and MS- probes, in three samples, could not be detected by a decrease in the signal of the MS- probe but was easily detected by the occurrence of a low but clear signal of the MS+ probe. The lower sensitivity of the MS- probes results mostly from the relatively high signal variation of the DS probes in cancer samples. The greater MLPA signal variation in cancer samples has been observed previously and results mostly from the genetic heterogeneity of cancer samples [20] and the lower quality and frequent degradation of DNA samples isolated from FFPE tissues [21–25].

### Amplification of EGFR, MET and ERBB2 and its relation to mutation frequency

**Fig 2:**
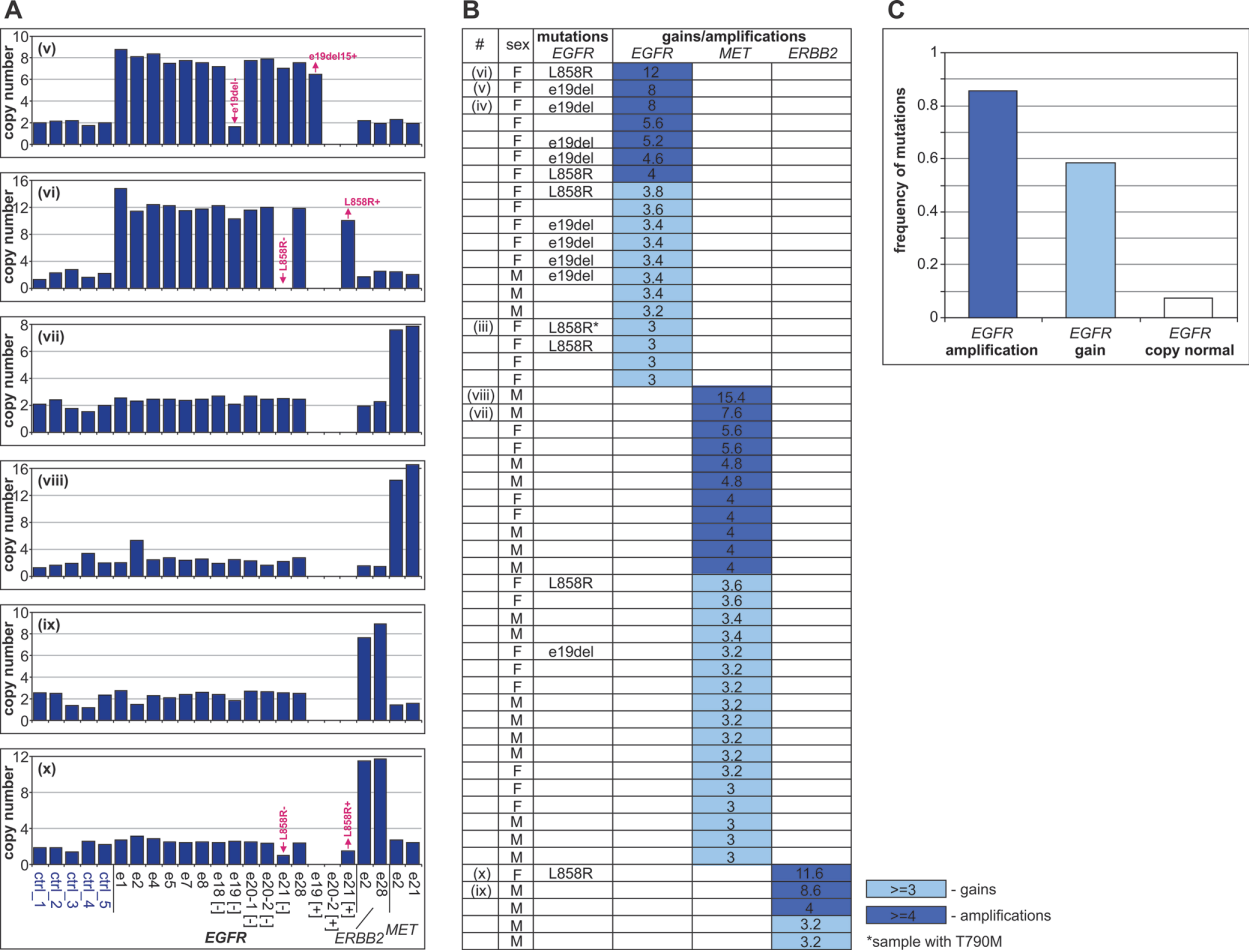
*EGFR*, *MET* and *ERBB2* amplifications in NSCLC samples. A) Examples of *EGFR* (samples v-vi), *MET* (samples vii-viii) and *ERBB2* (samples ix-x) amplification detected by the EGFRmut+ assay. B) Characteristics of samples with *EGFR*, *MET* and *ERBB2* gains/amplifications. The samples shown in Fig. 1D (ii-iii) and Fig. 2A (vi-x) are indicated in the first column. The sex and *EGFR* mutation status of each sample are indicated in the second and the third columns, respectively. Columns 4–6 show the copy number values of *EGFR*, *MET* and *ERBB2*, respectively. The dark blue cells indicate amplifications (copy number ≥4), and the light blue cells indicate gains (copy number 3–4). C) Frequency of *EGFR* mutations in samples with *EGFR* amplification, *EGFR* gain and normal *EGFR* copy number.

The additional advantage of the MLPA assay is that in addition to detecting mutations, it also provides information about a relative copy number of all analyzed sequences/probes. It allows a rough estimation of the fraction of detected mutations in analyzed samples and allows detection of copy number changes (amplifications) in the analyzed genes: *EGFR*, *MET* and *ERBB2*. Defining relative copy number 3–4 as a “gain” and ≥4 as an “amplification,” we identified 12 (5%), 17 (7%) and, 2 (1%) samples with gains and 7 (3%), 11 (5%) and, 3 (1%) samples with amplifications of *EGFR*, *MET*, and *ERBB2*, respectively (S1 Table and Fig. 2). The distribution of copy number amplitude was similar for all 3 genes, with the greatest amplifications exceeding 10 copies. As in case of the mutations, we observed a much higher frequency of *EGFR* gains/amplifications in women than in men (18% versus 2%, respectively; p<0.0001). A similar trend was not observed for *MET* (13% versus 11%, respectively), and a reversed but not significant trend was observed for *ERBB2* (1% versus 3%).

As shown in Fig. 2, the gains and amplifications of *EGFR*, *MET* and *ERBB2* were mutually exclusive. However, we observed very strong association between the occurrence of *EGFR* mutations and *EGFR* amplification (Chi square test for trend; p<0.0001). All but one cancer sample (90%) with *EGFR* amplification and 7 out of 12 samples (58%) with *EGFR* gain had an *EGFR* mutation.

A careful examination of the MLPA results with *EGFR* amplifications (see examples in Fig. 1 and Fig. 2) indicates that the signal decrease from the MS- probes and the signal increase of the MS+ probes (fraction of mutated copies) is approximately the same or even greater than the increase in *EGFR* copy number. This result suggests that all of the amplified copies of *EGFR* in the mutant samples contain the mutation (i.e., that only the mutant allele undergoes amplification) and that mutations occur as an early triggering event, making *EGFR* amplification beneficial for cancer. Note that all of the samples contain some amount of normal DNA, which may flatten the observed copy number changes and mask the effect in samples with a lower level of amplification.

This observation prompted us to sequence exons 18–21 (encoding the tyrosine kinase domain) in samples with *EGFR* gains/amplifications in which no known *EGFR* mutation was found. The sequencing analysis did not reveal any new sequence variants that could be oncogenic mutations or trigger *EGFR* amplification.

### Replication of EGFR copy number analysis (amplification detection) with the use of qPCR and ddPCR

To confirm the results of *EGFR* copy number analysis we reanalyzed a panel of cancer samples, including all samples with *EGFR* amplification, with the use of qPCR and ddPCR. Both methods are based on completely different principles than MLPA. The qPCR is a method utilizing real-time quantification of PCR product, most commonly used for verification of copy number variants, identified in both normal (non-cancer) and cancer samples (e.g. [26,27]). The ddPCR is a new quantification method, based on absolute counting of tested and reference DNA molecules, clonally amplified in thousands of water-in-oil droplet-reactions (emulsion PCR). The usefulness of ddPCR with the EvaGreen dsDNA-binding dye for precise copy-number estimation was recently demonstrated in several studies [18,19,28]. With the use of both qPCR and ddPCR, the signals of two test-amplicons, located in exon 2 and exon 18 of *EGFR*, were analyzed against the signal of control-amplicon (corresponding to MLPA probe ctrl_1). The analysis performed showed that results of both qPCR and ddPCR correlate very well with the relative copy number values, determined with the use of MLPA (correlation coefficient R = 0.941 and R = 0.927, respectively), and that the signals of samples with *EGFR* amplification were well separated from signals of samples without amplification (S1 Fig.). It has to be noted however that it cannot be excluded that some samples with borderline copy number values may be misclassified. Some discrepancies in the absolute signal values, determined by MLPA, and the reference methods may result from different sets of control regions used for normalization and from the fact that MLPA is a multiplex method and it measures the copy number in multiple points in a gene of interest. Similar results were obtained for *MET* and *ERBB2* (data not shown).

## Discussion

It is well known that the frequency of *EGFR* mutations differs substantially between human populations. The mutation frequency is highest in Asians (45–52%); lower in Europeans (24%), African Americans (20%) and Hispanics (17%); and lowest in White Australians (7%) ([11,29] and references within). Also among Europeans, different frequencies of *EGFR* mutations were reported: e.g. 17% in Spain [30] and 10% in southern Germany [31]. In this study, based on a comprehensive analysis of a substantial number of lung adenocarcinoma samples, we characterized and determined the frequency of *EGFR* mutations in Polish patients (12.1%). Our analysis showed a much higher (4.8x) frequency of *EGFR* mutations in women than in men (24% vs. 5%, respectively). We observed a similar trend for *EGFR* gains/amplifications, which showed even greater overrepresentation (9x) in women than in men (18% vs. 2%, respectively). Although the higher frequency of *EGFR* mutations in women than in men has been observed many times before, the overrepresentation of *EGFR* mutations in women observed in our study is, according to our knowledge, one of the highest reported so far (excluding the studies with a very small number of samples/mutations) ([11,29] and the references within). The information about population-specific characteristic and frequency of mutations are important epidemiological factors that may influence implementation of adequate diagnostic and treatment procedures optimal for particular population.

Molecular cancer diagnosticians address diversified heterogeneous tumor material on a daily basis. DNA is isolated from surgically resected tumors (fresh or FFPE) and from fine needle and endobronchial ultrasound transbronchial needle-aspirated cells. Each of these histological or cytological samples can have different and sometimes very low PTC, and they often show a substantial level of DNA degradation (especially the FFPE samples). Additionally, formalin fixation may damage DNA and lead to sequence modifications. All of these factors cause different types of artifacts that may lead to both false-positive and false-negative results.

In this study, we tested an EGFRmut+ MLPA assay (a standard-sensitivity method) as a second-tier method and compared our results with those of a well-validated commercial RT-PCR assay (a high-sensitivity method). These two methods are based on completely different principles. In RT-PCR, mutation detection is based on the hybridization of TaqMan mutation-specific probes, but in MLPA, mutation detection is based on the ligation and subsequent amplification of wild type or mutation-specific probes. Our results indicate that EGFRmut+ is a robust assay that exhibits high reproducibility, specificity and sensitivity. A small number of undetected mutations were either not covered by the MLPA probes (n = 2) or occurred in a sample with a low PTC (n = 1). The EGFRmut+ assay allowed us to detect mutations in all types of samples (cytological and histological; fresh frozen and FFPA) and allowed us to detect mutations in samples with different PTCs (20–90%). However, it must be noted that MS+ probes allow more robust mutation detection than MS- probes do and that the quality of MLPA results is generally lower for cancer samples than for germ-line DNA samples. The lower quality of cancer MLPA results is due to the aforementioned characteristics of cancer samples and has been reported and discussed previously [23–25,32]. Additional factors that make the MLPA assay attractive as a second-tier *EGFR* mutation detection method are the low amount of DNA (50–100 ng) required for analysis (no additional sample extraction or preparation is required, and the same DNA sample can be used for both RT-PCR and MLPA analysis), a short turn-around time (<2 days), and low cost (roughly $5 plus the initial cost of probe synthesis, approximately $3,000). In the case of routinely used assays, the cost of probe synthesis may be ignored because the quantity of the synthesized probes is sufficient for hundreds of thousands of analyses.

In addition to *EGFR* mutation detection, the EGFRmut+ assay also allows the detection of *EGFR*, *MET* and *ERBB2* amplification. Although the data are not conclusive, it has been suggested that *EGFR* amplification may be an indicator or modifier of sensitivity to TKI treatment (reviewed in [11]). It also has been suggested that the amplification of either *MET* or *ERBB2* may be an alternative mechanism underlying acquired resistance to TKI treatment (reviewed in [11]). In our study, we found that *EGFR*, *MET* and *ERBB2* gains and amplifications are mutually exclusive and that *MET* and *ERBB2* gains/amplifications rarely co-occur with *EGFR* mutations. Only one *EGFR* mutation co-occurs with an *ERBB2* amplification, and two *EGFR* mutations co-occur with a gain of *MET*. These data suggest a rather independent occurrence of *EGFR* mutations and *MET*/*ERBB2* amplifications and may argue against the role of *MET*/*ERBB2* amplification as a mechanism of acquired resistance to TKIs. However, to investigate this observation further, analysis of lung cancer samples after treatment with TKI should be performed. On the other hand, we observed a very strong correlation between *EGFR* gains/amplifications and the occurrence of *EGFR* mutations. All but one sample with an *EGFR* amplification also had an *EGFR* mutation. This suggests that activating *EGFR* mutations are the triggers of *EGFR* amplifications; cases of *EGFR* amplification in which no mutations were found may therefore be triggered by mutations that are not covered by standard *EGFR* tests. Such mutations may occur outside the tyrosine kinase domain and in non-coding regulatory sequences. Although the co-occurrence of *EGFR* mutations and amplifications has been observed before, it is typically much less pronounced than was observed in our study (e.g., the recent whole-genome analysis of 183 lung adenocarcinomas with the use of massively parallel sequencing [33]). These discrepancies may result from the different methods used for *EGFR* copy number determination.

In our laboratory, we routinely analyze somatic *EGFR* mutations in NSCLC samples from a large area of Poland (mostly central Poland) with a commercial RT-PCR test (EGFR-RT52). As it was suggested in recommendation for *EGFR* testing [11], we attempted to set up a second-tier method for the detection of somatic *EGFR* mutations. For this purpose, we selected an MLPA-based assay (EGFRmut+), and the results of this assay were validated against those of RT-PCR. Our study showed a very high concordance between the results of these two methods and thus confirmed both the robustness of mutation detection by MLPA assays and its usefulness as second-tier method for *EGFR* testing. Two additional advantages of the MLPA assay are its ability to carry out copy number (amplification) analysis and the potential to estimate the relative proportion of the mutated allele in a sample. A similar strategy of analysis and a similar MLPA-based assay may be developed and used for the analysis of other important oncogenes in other types of cancer.

## Supporting Information

S1 Fig Replication of MLPA results of *EGFR* copy number analysis with the use of qPCR and ddPCR. Scatter plots showing correlation between relative copy number signals determined by MLPA (x-axis) and relative signals determined either by qPCR (A), or ddPCR (B) (y-axis). The values depicted on y-axis represent averaged signals, measured in exon 2 and 18 of *EGFR* (see Materials and Methods). The blue, light-blue and white dots indicate samples with *EGFR* amplification (N = 7), gain (N = 3) and normal copy number (N = 6), respectively. The trend line and correlation coefficient are indicated on each graph.
(PDF)

S1 Table Mutations and Copy Number Alterations Detected in NSCLC Samples. (XLS)
