# Supplementary material for: The Use of a Two-Tiered Testing Strategy for the Simultaneous Detection of Small EGFR Mutations and EGFR Amplification in Lung Cancer
Source: PLoS One. 2015 Feb 26;10(2):e0117983. doi: 10.1371/journal.pone.0117983 (PMC4342230; doi:10.1371/journal.pone.0117983)
Supplement: S1 Fig — Scatter plots showing correlation between relative copy number signals determined by MLPA (x-axis) and relative signals determined either by qPCR (A), or ddPCR (B) (y-axis). The values depicted on y-axis represent averaged signals, measured in exon 2 and 18 of EGFR (see Materials and Methods). The blue, light-blue and white dots indicate samples with EGFR amplification (N = 7), gain (N = 3) and normal copy number (N = 6), respectively. The trend line and correlation coefficient are indicated on each graph. (PDF) [file pone.0117983.s001.pdf]

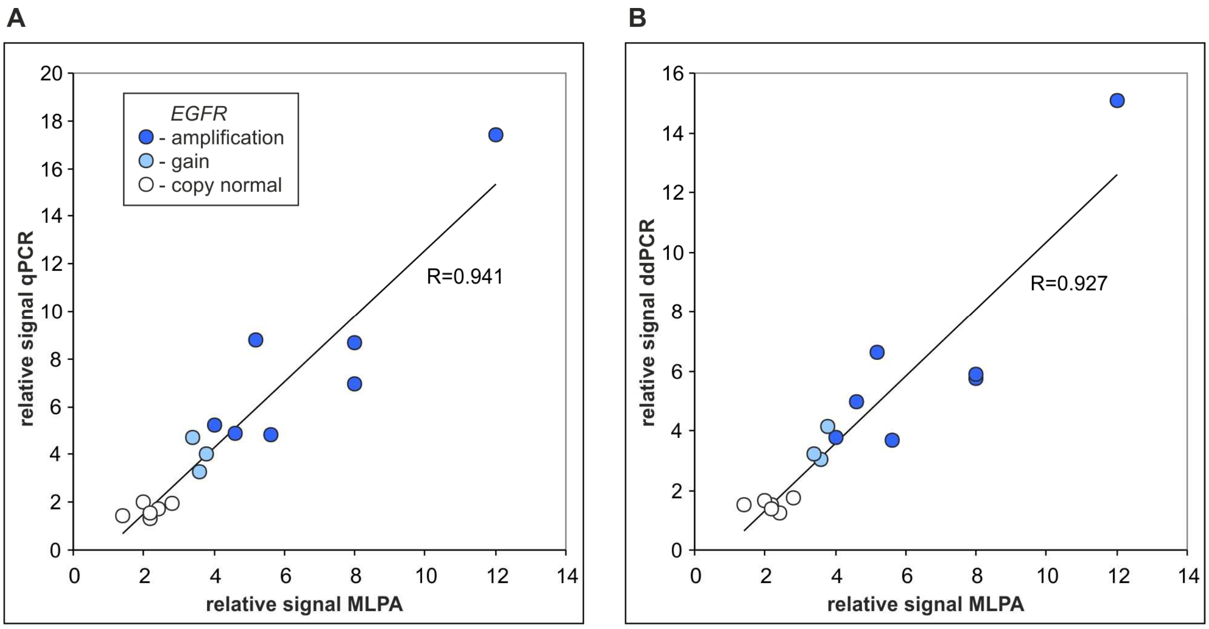

S1 Figure. Replication of MLPA results of *EGFR* copy number analysis with the use of qPCR and ddPCR. Scatter plots showing correlation between relative copy number signals determined by MLPA (x-axis) and relative signals determined either by qPCR (A), or ddPCR (B) (y-axis). The values depicted on y-axis represent averaged signals, measured in exon 2 and 18 of *EGFR* (see Materials and Methods). The blue, light-blue and white dots indicate samples with *EGFR* amplification (N=7), gain (N=3) and normal copy number (N=6), respectively. The trend line and correlation coefficient are indicated on each graph. (PDF)
